# Long-lasting blocking of interoceptive effects of cocaine by a highly efficient cocaine hydrolase in rats

**DOI:** 10.1038/s41598-023-50678-0

**Published:** 2024-01-09

**Authors:** Huimei Wei, Johnathan E. LeSaint, Zhenyu Jin, Chang-Guo Zhan, Fang Zheng

**Affiliations:** 1 Molecular Modeling and Biopharmaceutical Center, College of Pharmacy, University of Kentucky, 789 South Limestone Street, Lexington, KY 40536 USA; 2 Department of Pharmaceutical Sciences, College of Pharmacy, University of Kentucky, 789 South Limestone Street, Lexington, KY 40536 USA

**Keywords:** Recombinant protein therapy, Addiction

## Abstract

Cocaine dependence is a serious world-wide public health problem without an FDA-approved pharmacotherapy. We recently designed and discovered a highly efficient long-acting cocaine hydrolase CocH5-Fc(M6). The present study examined the effectiveness and duration of CocH5-Fc(M6) in blocking interoceptive effects of cocaine by performing cocaine discrimination tests in rats, demonstrating that the duration of CocH5-Fc(M6) in blocking cocaine discrimination was dependent on cocaine dose and CocH5-Fc(M6) plasma concentration. Particularly, a dose of 3 mg/kg CocH5-Fc(M6) effectively attenuated discriminative stimulus effects of 10 mg/kg cocaine, cumulative doses of 10 and 32 mg/kg cocaine, and cumulative doses of 10, 32 and 56 mg/kg cocaine by ≥ 20% for 41, 19, and 10 days, and completely blocked the discriminative stimulus effects for 30, 13, and 5 days with corresponding threshold plasma CocH5-Fc(M6) concentrations of 15.9, 72.2, and 221 nM, respectively, under which blood cocaine concentration was negligible. Additionally, based on the data obtained, cocaine discrimination model is more sensitive than the locomotor activity to reveal cocaine effects and that CocH5-Fc(M6) itself has no long-term toxicity regarding behavioral activities such as lever pressing and food consumption in rats, further demonstrating that CocH5-Fc(M6) has the desired properties as a promising therapeutic candidate for prevenance of cocaine dependence.

## Introduction

Cocaine use disorder (CUD) remains a significant social, economic, and public health burden world-wide. An estimated 20 million people had used cocaine globally and around 6.9 million users located in North America in 2019^1^. The health harms caused by substance use disorders have further deteriorated during the COVID-19 crisis^2–4^. The number of cocaine users and overdose deaths have risen steadily in the past few years^5–7^. Nevertheless, only ~ 19% of people with CUD received clinical management (behavioral therapy mainly)^8–10^. Since there is no FDA approved pharmacotherapy for CUD, a lot of people who suffer from CUD remain without appropriate care.

The conventional approach to develop small-molecule compounds reversing cocaine effects in the central nervous system (CNS) may result in disrupting normal CNS functions and induce adverse effects as cocaine utilizes our brain’s normal circuits and physiology to exert its functions^11,12^. Other approaches such as anti-cocaine vaccines and monoclonal antibodies are limited by the capacity for binding larger number of cocaine molecules than available binding sites of the antibodies^13–15^. To develop a truly effective therapeutics for CUD, through development and use of novel computational enzyme redesign methodologies^16–22^, our lab successfully designed and discovered a series of cocaine hydrolases (CocHs)^21–43^ by introducing mutations into human butyrylcholinesterase (hBChE) which originally has a low catalytic activity against cocaine at physiological condition (*k*_cat_ = 4.1 min^−1^; *K*_M_ = 4.5 µM)^44–48^. These mutants of hBChE are able to hydrolyze and eliminate cocaine at the benzoyl ester moiety to produce physiologically inactive metabolites in plasma with at least 1000-fold improved catalytic efficiency against cocaine compared to wild-type hBChE. Our first CocH, denoted as CocH1 (i.e. A199S/S287G/A328W/Y332G mutant of hBChE: *k*_cat_ = 3060 min^−1^; *K*_M_ = 3.1 µM)^48^, fused with human serum albumin (HSA) (known as TV-1380, Albu-CocH, Albu-CocH, Albu-CocH1 or AlbuBChE in the literature) has been widely tested^38,42,45,49–54^, including three Phase I clinical trials and a Phase II clinical trial to develop a therapeutic for facilitating abstinence in cocaine-dependent subjects. The results of Phases I and II clinical trials conveyed positive outcomes regarding safety and efficacy of TV-1380. Particularly, the Phase II trial^51^ revealed that once-weekly dosing of TV-1380 significantly and dose-dependently decreased cocaine intake in the treatment groups compared to the placebo group. However, the practical clinical development with TV-1380 is limited by the relatively lower catalytic efficiency of CocH1 for cocaine hydrolysis and the shorter biological half-life of TV-1380 (t_1/2_ = 8 h in rats and t_1/2_ = 43 − 77 h in humans) for a once-weekly dosing regimen^49–51^, which argued for development of improved enzymes with greater catalytic activity against cocaine and longer half-life in plasma^51^. To address this issue, we reengineered BChE to further increase catalytic activity against cocaine^55^. Among the discovered BChE mutants, the most efficient one is CocH5 (A199S/F227A/P285A/S287G/A328W/Y332G mutant of hBChE: *k*_cat_ = 14,600 min^−1^; *K*_M_ = 3.65 µM)^47^ with ~ 4000-fold improved catalytic efficiency against cocaine compared to wild-type BChE.

To design a long-acting form of CocHs, CocHs were linked to the N-terminus of human immunoglobulin G1 (IgG1) Fc fragment, instead of HSA, to produce Fc-fused CocH proteins (denoted as CocH-Fc)^39,43,55,56^. Furthermore, we designed a Fc mutant, denoted as Fc(M6) for convenience, by introducing A1V/M38Y/S40T/T42E/D142E/L144M mutation in the Fc portion of IgG1, which led to largely prolonged biological half-life of the CocHs^56^. For our most efficient CocH, unfused CocH5 only has an elimination half-life of ∼6 h (t_1/2_ = ~ 6 h) in rats, while after fusing with Fc(M6), CocH5-Fc(M6) has the longest elimination half-life (t_1/2_ = 229 ± 5 h)^57^ in rodents compared to all other CocHs. Notably, protein fusion with Fc(M6) does not significantly alter the catalytic efficiency of the Fc-fusion protein produced against cocaine. CocH5-Fc(M6), with *k*_cat_ = 13,800 min^−1^ and *K*_M_ = 3.89 µM^57^, represents the currently most efficient long-acting CocH against cocaine.

In our previous studies, it was demonstrated that CocH5-Fc(M6) rapidly hydrolyzed the cocaine in blood and powerfully rescued rats from a lethal dose of cocaine for cocaine overdose treatment^57^; it was also demonstrated that CocH5-Fc(M6) effectively blocked 20 mg/kg cocaine (IP)-induced dopamine transporter trafficking (associated with cocaine dependence) and hyperactivity with repeated cocaine exposures by maintaining a plasma CocH5-Fc(M6) concentration ≥ 58.7 ± 2.9 nM in rats which allowed the CNS to recover from the cocaine-caused malfunction^57–60^. To further investigate the ability of such a potent enzyme for prevention of the central effects associated with cocaine addiction, in the present study, we employed the drug discrimination (DD) procedure to examine the interventional role of CocH5-Fc(M6) on interoceptive effects of cocaine. In drug abuse research, the drug discrimination (DD) paradigm is designed to detect discriminative stimulus property of a psychoactive drug that is considered to be an important factor affecting drug-taking and drug abuse-related reinforcing behaviors^61–64^. In the DD model, a subject has to recognize or perceive a particular drug state (drug/nondrug condition), choose a correct response, and then receive reinforcement (or reward)^65,66^. Because of its pharmacological sensitivity and specificity on the effect of a training drug, the DD technique is also an important component to study drug-abuse behavior besides self-administration and conditioned place-preference procedures^67,68^. The DD paradigm has also been widely used to evaluate new therapeutic agents to antagonize the discriminative stimulus effects of abused substances^69–72^. Therefore, the DD tests with various doses of the enzyme and cocaine allowed us to determine the effectiveness and duration of CocH5-Fc(M6) in blocking the discriminative stimulus effects of cocaine in rats.

## Results

### Impact of CocH5-Fc(M6) on response for food pellets

Before conducting more extensive DD tests to determine how CocH5-Fc(M6) affects discriminative stimulus effects of cocaine, we wanted to know whether CocH5-Fc(M6) affects food self-administration. 3 mg/kg CocH5-Fc(M6) was intravenously administered to rats. All rats obtained the maximum number of rewards (40 food pellets) in all pre- and post-treatment testing sessions which were conducted intermittently up to 32 days (i.e., on days 0, 2, 4, 6, 8, 12, 16, 20, 24, 28, and 32) after enzyme treatment. As far as the response rate was concerned, there was no significant difference detected in repeated one-way ANOVA analysis (F(3.667, 25.67) = 0.8274; *p* = 0.5113). The study confirmed that CocH5-Fc(M6) itself had no adverse effects on the motivation for food consumption in rats.

### Cocaine dose–response curve

**Figure 1:**
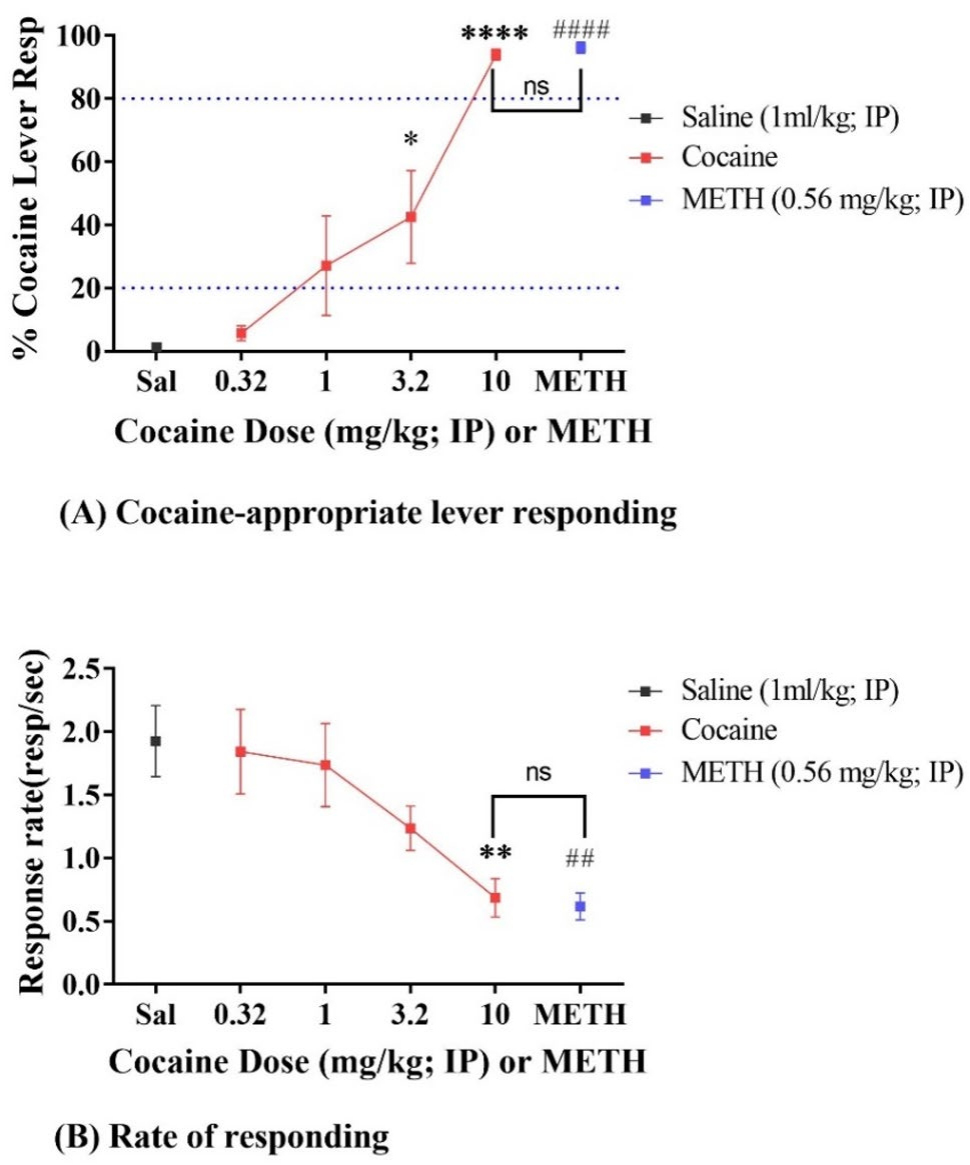
Dose–response curve of cocaine during cumulative dosing test (n = 8). (**A**) Cocaine-appropriate lever responding during dose–response curve test; (**B**) Rate of responding during cocaine dose–response curve test. Cocaine was administered in a cumulative dosing procedure. Data are presented as mean ± SEM and analyzed by one-way ANOVA with post hoc tests: *p < 0.05, **p < 0.01, and ****p < 0.0001 for significant difference from cocaine to saline; METH to saline and METH to Cocaine: ^##^*p* < 0.01 and ^####^*p* < 0.0001 for significant difference from METH to cocaine or saline; ns for no significant difference detected.

**Figure 2:**
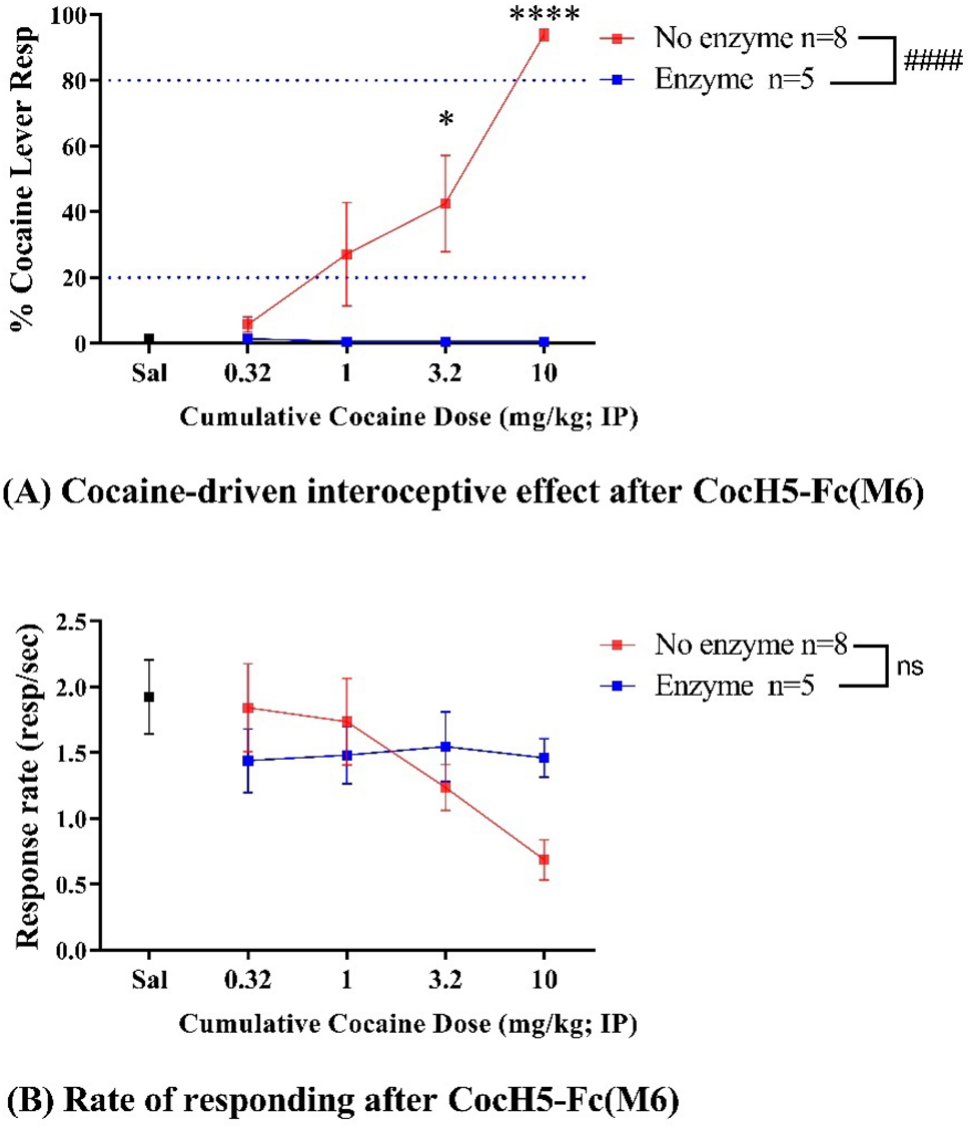
The impact of CocH5-Fc(M6) on cocaine dose–response curve. (**A**) Cocaine-driven interoceptive effects of cocaine with and without enzyme treatment; (**B**) Rate of responding during the test. Cocaine was administered in a cumulative dosing procedure. A low dose of enzyme (0.3 mg/kg) was given 4 h before the first component of the session by intravenous injection. Data are presented as mean ± SEM and are analyzed by two-way ANOVA: ^####^*p* < 0.0001 for significant difference between the enzyme-treated and untreated groups; ns for no significant difference detected. Following the two-way ANOVA, post hoc test was used to compare cocaine-driven interoceptive effects of 3.2 mg/kg or 10 mg/kg cocaine with and without enzyme treatment: *p < 0.05 and ****p < 0.0001.

Before determining how CocH5-Fc(M6) affects cocaine discrimination in rats, we tested cocaine discrimination in rats with a series of cocaine doses (0.32, 1.0, 3.2, and 10 mg/kg). After rats acquired stable discriminative responding between cocaine and saline, a cocaine dose–response curve was examined with 0.32, 1.0, 3.2, and 10 mg/kg cocaine in a cumulative dosing procedure on the same day. With the DD test session of 25 min, one dose of cocaine injection was followed by another with 30 min in between. According to Figs. 1 and 2, the discriminative stimulus effect from 0.32, 1 or 3.2 mg/kg cocaine was either imperceptible or lasting less than 30 min. Thus, the discriminative effects from the DD test with either of the four cocaine doses used in the dose response testing are independent without intervention with those from other doses. 0.56 mg/kg methamphetamine (METH) was tested another day to demonstrate its ability to substitute cocaine discrimination stimulus. As shown in Fig. 1, with increasing the dose, cocaine produced cocaine-driven interoceptive effects in a dose-dependent manner (F(4, 35) = 14.65; *p* < 0.0001) according to one-way ANOVA analysis. The post hoc tests showed that 0.32 and 1 mg/kg cocaine induced non-significant cocaine lever responding (*p* = 0.9965 for 0.32 mg/kg; *p* = 0.1790 for 1 mg/kg), while 3.2 and 10 mg/kg cocaine led to significant cocaine lever responding compared to saline (*p* = 0.0108 for 3.2 mg/kg; *p* < 0.0001 for 10 mg/kg), so did 0.56 mg/kg METH (*p* < 0.0001). Both 10 mg/kg cocaine and 0.56 mg/kg METH led to 94% and 96% of overall responding occurring on the cocaine-lever respectively without significant difference (*p* > 0.9999). When it came to the response rate during this test, cocaine produced a dose-dependent decrease in the response rate (F (4, 35) = 3.850; *p* = 0.0107); similar dose-dependent decrease in the response rate was also noted previously^73^. Especially, 10 mg/kg cocaine slowed down the response rate significantly based on the post hoc analysis (*p* = 0.0079) compared to saline, and the same is true for 0.56 mg/kg METH (*p* = 0.0013). There is no significant difference in the response rate between 10 mg/kg cocaine and 0.56 mg/kg METH (*p* > 0.9999). Thus, cocaine produced a dose-dependent increase in the selection of the cocaine-appropriate lever (generalization to the discriminative stimulus effects of cocaine), dose-dependent decrease in the response rate, and 0.56 mg/kg METH was able to fully substitute 10 mg/kg cocaine in the drug discrimination test.

### Impact of CocH5-Fc(M6) on cocaine-driven interoceptive effects

To know how CocH5-Fc(M6) affects cocaine discrimination in rats, we first tested a low dose (0.3 mg/kg) of CocH5-Fc(M6) for its effects on cocaine discrimination with the cumulative dosing procedure of 0.32, 1.0, 3.2 and 10 mg/kg cocaine described previously. According to two-way ANOVA analysis, 0.3 mg/kg CocH5-Fc(M6) was able to block the cocaine-driven interoceptive effects of cocaine significantly (F(1, 44) = 35.09; *p* < 0.0001) and completely in the cumulative dosing procedure to the level that the rats could not perceive the stimulus of cocaine at all even with the dose of 10 mg/kg cocaine, as indicated in Fig. 2. The post hoc test showed that the enzyme depleted discriminative effects produced by 3.2 and 10 mg/kg cocaine profoundly compared to the control without enzyme treatment (with *p* = 0.0181 and *p* < 0.0001, respectively). No significant difference was detected in the response rate between the enzyme group and the saline control (no cocaine) in two-way ANOVA analysis (F(1, 44) = 0.3277; *p* = 0.5700). In addition, 10 mg/kg cocaine after the enzyme pretreatment did not suppress the response rate anymore, instead, produced a response rate very close to saline (~ 1.5 responses/s), much higher than the response rate produced by 10 mg/kg cocaine without enzyme (~ 0.69 responses/s). The data suggests that rats did not sense any trait of 10 mg/kg cocaine after pre-treated with a low dose of CocH5-Fc(M6) and, instead, they exclusively responded on saline-appropriate lever in this test. These results inspired us to extend our study to determine the long-term effects of CocH5-Fc(M6) through further DD tests.

### Long-term impact of CocH5-Fc(M6) on discriminative stimulus effects of cocaine

**Figure 3:**
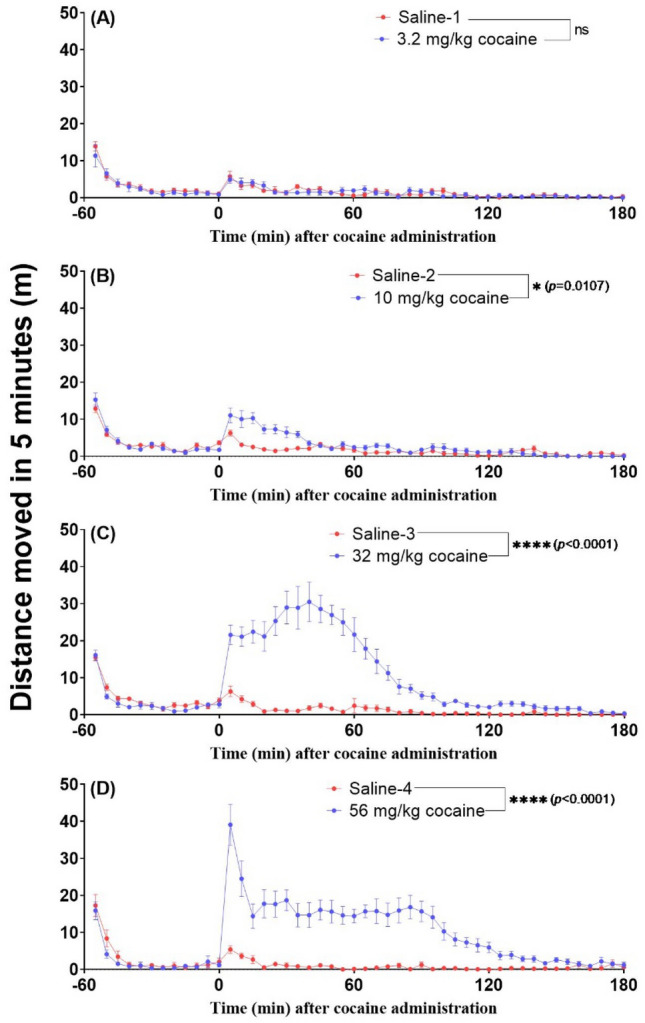
Cocaine-induced hyperactivity. Cocaine doses: (**A**) 3.2 mg/kg (n = 8), (**B**) 10 mg/kg (n = 8), (**C**) 32 mg/kg (n = 8), and (**D**) 56 mg/kg (n = 7). The rats were first allowed to acclimate to the testing chambers for 60 min before intraperitoneal administration of cocaine or saline. The locomotor activity data are plotted as the mean ± SEM meters travelled in 5-min bin during locomotor activity session for 4 h. Two-way repeated measure ANOVA revealed that 10, 32, and 56 mg/kg cocaine induced significant hyperactivity than saline with *p* level marked in the figure. 56 mg/kg cocaine led to significant toxicity such as convulsion after the cocaine injection in addition to hyperactivity; one rat died after administration of 56 mg/kg cocaine and, hence, only 7 rats completed the 56 mg/kg session.

**Figure 4:**
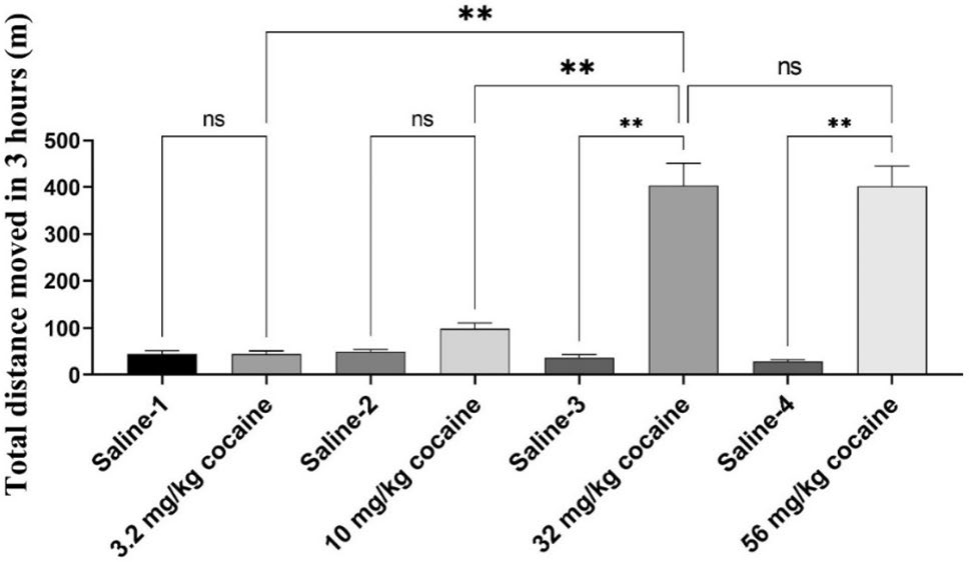
Total distance moved in 3 h after cocaine injection. Cocaine doses: 3.2 mg/kg (n = 8), 10 mg/kg (n = 8), 32 mg/kg (n = 8), and 56 mg/kg (n = 7). One-way ANOVA showed that 32 mg/kg cocaine caused much stronger hyperactivity compared to 3.2 and 10 mg/kg cocaine. Particularly, 56 mg/kg cocaine led to significant toxicity such as convulsion (that stated at 5–15 min after the 56 mg/kg cocaine injection) in addition to hyperactivity and could be lethal (one rat died at 13 min after administered with 56 mg/kg cocaine and, hence, only 7 rats completed the 56 mg/kg session).

**Figure 5:**
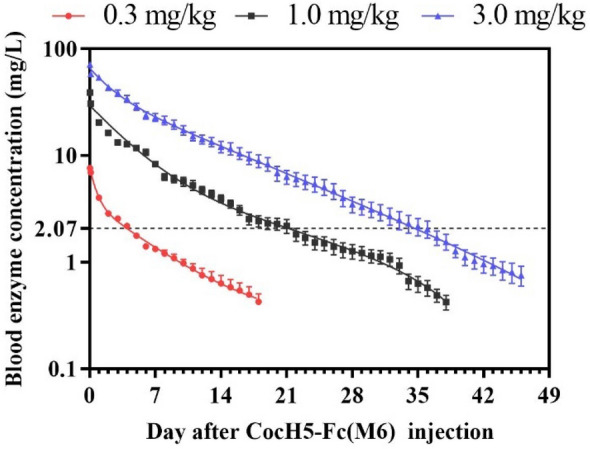
The time-dependent mean plasma concentrations of CocH5-Fc(M6) at various doses in cocaine discrimination tests: 0.3 mg/kg (n = 7), 1 mg/kg (n = 6), and 3 mg/kg (n = 7). The horizontal line indicated the average threshold plasma concentration for various doses of CocH5-Fc(M6) required to completely block the discriminative stimulus effect of 10 mg/kg cocaine in rats.

**Figure 6:**
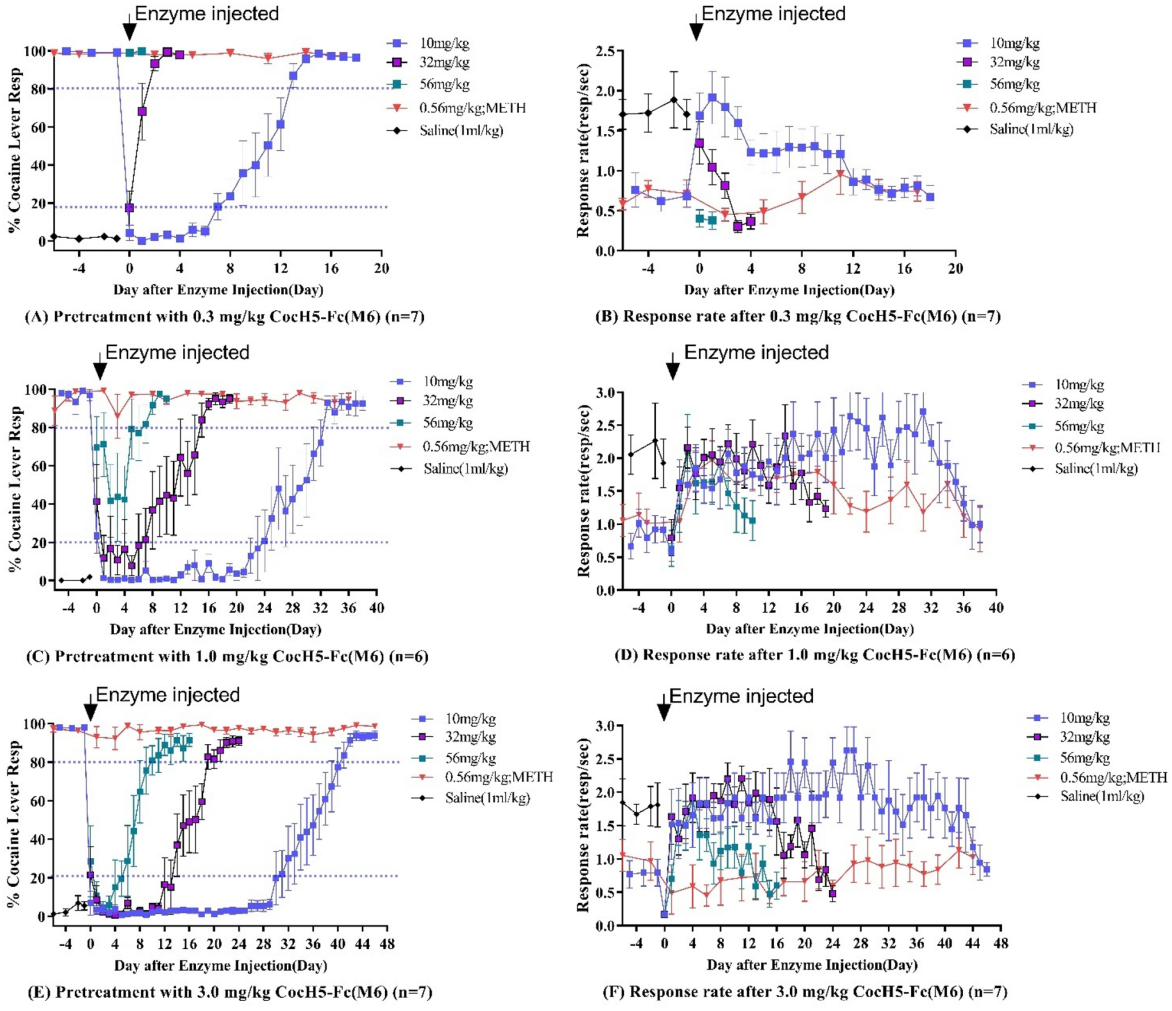
The long-term cocaine-driven interoceptive effect following pretreatment with CocH5-Fc-(M6) in cocaine discrimination tests. A single dose of CocH5-Fc(M6) (0.3, 1.0, or 3.0 mg/kg; IV) was injected 4 h before the first component of the DD test session on day 0 in three different groups of rats associated with the enzyme dose of 0.3 mg/kg (n = 7), 1 mg/kg (n = 6), or 3 mg/kg (n = 7). Cocaine discrimination tests with the cumulative doses of 10, 32, and 56 mg/kg cocaine (IP) were carried out daily until the enzyme could not block the discriminative stimulus effect of 10 mg/kg cocaine. The black arrow indicated the time of CocH5-Fc-(M6) injection.

To evaluate the duration of CocH5-Fc(M6)’s effects, a long-term drug discrimination study was conducted. A single dose of the enzyme (low-0.3 mg/kg, medium-1.0 mg/kg, or high-3.0 mg/kg) was intravenously administered on day 0 as pretreatment before cocaine discrimination daily tests with cumulative doses of 10, 32, and 56 mg/kg, IP. The doses of 32 and 56 mg/kg cocaine were added to further challenge the ability of the enzyme for preventing cocaine effects. As shown in Figs. 2, 3 and 4, 3.2 mg/kg cocaine significantly produced an average of 42.6% drug-appropriate responding (Fig. 2) but could not bring about any hyperactivity activity increase (Fig. 3) in rats, indicating that the discriminative effect of cocaine is more sensitive than the cocaine-induced hyperactivity; 10 mg/kg cocaine was sufficient to produce cocaine-driven interoceptive effects in the DD test and sufficient for rats perceiving cocaine 40 min once cocaine was injected in locomotor activity test; intraperitoneal administration of 32 mg/kg cocaine caused much stronger hyperactivity lasting more than 2 h compared to 3.2 and 10 mg/kg cocaine, whereas 56 mg/kg cocaine resulted in significant toxicity (such as convulsion) in addition to the hyperactivity for more than 2 h and was even lethal (one of 8 rats died at 13 min after receiving 56 mg/kg cocaine, IP). The toxicity caused by cocaine, particularly the convulsion (that started at 5–15 min after 56 mg/kg cocaine injection), significantly slowed down the hyperactivity. Therefore, unlike the lower-dose cocaine of 0.32, 1 or 3.2 mg/kg, during the DD test with the cumulative dosing procedure of 10, 32 and 56 mg/kg cocaine, the drug effects caused by one dose were cumulative effects of the dose plus the previous doses of cocaine. Figure 5 showed the time-dependent mean plasma concentration of CocH5-Fc(M6) used in the cocaine discrimination tests, indicating that CocH5-Fc(M6) has a linear first-order elimination pharmacokinetic property. As shown in Fig. 6, CocH5-Fc(M6) dose-dependently blocked the discriminative stimulus effects induced by 10 mg/kg cocaine, cumulative doses (10 and 32 mg/kg) of cocaine, and cumulative doses (10, 32, and 56 mg/kg) of cocaine for different periods of time (as summarized in Table 1). To calculate the duration of the blocking effects of the enzyme, the last day for the enzyme completely blocking the discriminative stimulus effects of cocaine was counted as the day when cocaine produced less than 20% of the overall responding, occurring on the cocaine-appropriate lever after the enzyme injection; the first day of full generalization to the discriminative stimulus effects of cocaine was counted as the day when cocaine produced more than 80% of overall responding occurring on the cocaine-appropriate lever during any component (10 or 32 or 56 mg/kg cocaine) of the DD session, which also suggested that the enzyme could not block cocaine discriminative stimulus effects any longer starting from that time point.

**Table 1:** Long-lasting blocking of the discriminative stimulus effects of cocaine by CocH5-Fc(M6).

% Cocaine-appropriate responding		Number of days for complete blocking			Number of days for full generalization		
Cumulative cocaine doses of		10 mg/kg	10,32 mg/kg	10,32,56 mg/kg	10 mg/kg	10,32 mg/kg	10,32,56 mg/kg
Dose of CocH5-Fc-(M6)	0.3 mg/kg	6	1	na	13	2	na
	1.0 mg/kg	23	6	na	33	15	7
	3.0 mg/kg	30	13	5	41	19	10
Average threshold plasma concentration (mg/L)		2.07	9.38	28.7	0.88	4.9	12.8
Average threshold plasma concentration (nM)		15.9	72.2	221	6.8	37.7	98.5

The duration (days) of completely blocking (less than 20% cocaine-appropriate lever responding) and full generalization (more than 80% cocaine-appropriate lever responding) to the discriminative stimulus effects of cocaine following the enzyme pretreatment were listed. Cocaine was administered cumulatively at doses 10, 32, and 56 mg/kg in each session of DD test. Average threshold plasma enzyme concentrations for the three doses of CocH5-Fc(M6) were included as well in both mg/L and nM. Abbreviation: na, not applicable.

For the discriminative effect produced by training dose of 10 mg/kg cocaine, 0.3 mg/kg CocH5-Fc(M6) was able to completely blocking the discriminative effect for 6 days and continued to significantly suppress the cocaine effect for 13 days (Fig. 6A); 1.0 mg/kg CocH5-Fc(M6) completely blocked the discriminative effect for 23 days and continued to suppress the effect for 33 days (Fig. 6C); 3.0 mg/kg CocH5-Fc(M6) completely blocked the discriminative effect for 30 days and continued to suppress the effect for 41 days (Fig. 6E). The average threshold plasma concentration of CocH5-Fc(M6) for completely blocking the discriminative stimulus effect of 10 mg/kg cocaine in rats was around 2.07 mg/L or 15.9 nM and continued to significantly suppress the cocaine effect when the plasma enzyme concentration [E] > 0.88 mg/L or 6.8 nM. Accordingly, the threshold plasma concentrations of CocH5-Fc(M6) for completely blocking the discriminative stimulus effects of cumulative doses of 10 and 32 mg/kg cocaine and cumulative doses of 10, 32, and 56 mg/kg cocaine in rats were about 9.38 and 28.7 mg/L or ~ 72.2 and ~ 221 nM, respectively. The duration of the CocH5-Fc(M6)’s effects against the discriminative stimulus effects of cocaine was dependent upon the cocaine dose used in the DD tests and the plasma concentration of CocH5-Fc(M6). For example, 3 mg/kg CocH5-Fc(M6) led to nearly exclusive responding on the saline-appropriate lever at discrimination with cumulative doses of 10, 32, and 56 mg/kg cocaine, cumulative doses of 10 and 32 mg/kg cocaine, and 10 mg/kg cocaine for 5, 13, and 30 respectively. In other words, a modest dose of 3 mg/kg CocH5-Fc(M6) completely blocked the discriminative effects induced by the cumulative doses of 10, 32, and 56 mg/kg cocaine, cumulative doses of 10 and 32 mg/kg cocaine, and 10 mg/kg cocaine for 5, 13, and 30 days, respectively. All the results, including those from the lower doses of CocH5-Fc(M6) discussed above, are summarized in Table 1.

When it came to response rate in this test, CocH5-Fc(M6) increased response rate of cocaine to the level of response rate resulting from saline (~ 2 resp./s) since rats exclusively responded on the saline-appropriate lever at three different cumulative doses (10, 32, and 56 mg/kg) of cocaine when the enzyme concentrations were high enough to completely block the discriminative effects of cocaine (Fig. 6B,D,F). Then the response rate gradually decreased as the plasma enzyme concentration decreased day by day. Eventually, the response rate returned to baseline which was produced by training dose of 10 mg/kg cocaine (~ 0.5 resp./s). All cocaine-lever responding percentage data were reversely correlated overall with all the response rate data. In this study, 0.56 mg/kg METH enabled us to fully substitute the discriminative effects of 10 mg/kg cocaine and exerted legitimate function as positive control in terms of both lever responding and response rate (Fig. 6).

### CocH5-Fc(M6) accelerated cocaine metabolism

**Figure 7:**
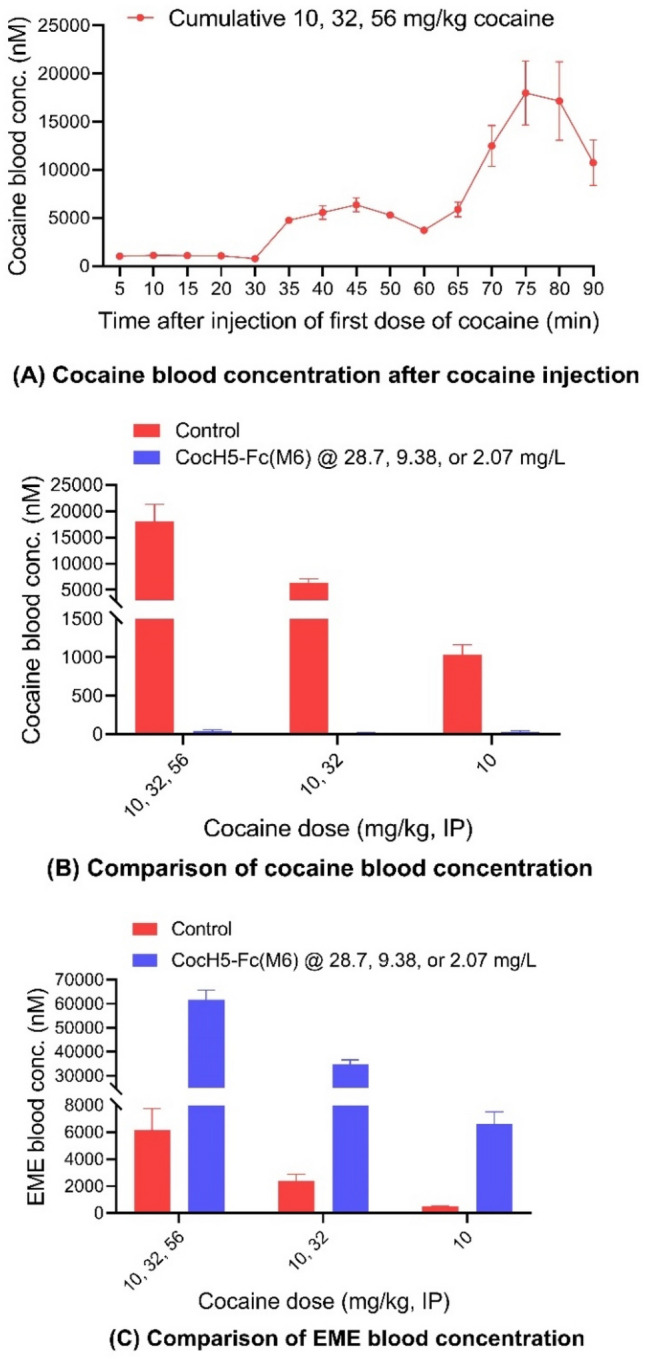
The blood concentrations of cocaine and EME in rats after receiving cumulative 10, 32, and 56 mg/kg cocaine (IP), cumulative 10 and 32 mg/kg cocaine (IP), or 10 mg/kg cocaine (IP). Rats (n = 6 for the control group or n = 4/group with the enzyme treatment) were injected with 10 mg/kg cocaine at time zero (0 min time point), 32 mg/kg cocaine at 30 min time point for the groups receiving the dose of 32 mg/kg cocaine, and 56 mg/kg at 60 min time point for the cumulative doses of 10, 32, and 56 mg/kg cocaine groups*.* In CocH5-Fc(M6)-treated rats, CocH5-Fc(M6) was presented at the threshold level ~ 28.7 mg/L for cumulative doses of 10, 32, and 56 mg/kg cocaine group, ~ 9.38 mg/L for cumulative doses of 10 and 32 mg/kg cocaine group, and ~ 2.07 mg/L for 10 mg/kg cocaine group. (**A**) Time course of blood cocaine concentrations in control group of rats without CocH5-Fc-(M6), showing the peaks at 5 min, 45 min (or 15 min after 32 mg/kg cocaine), and 75 min (or 15 min after 56 mg/kg cocaine). Note that n = 4 for all time points, except for the 90 min time point (when n = 2, as only two rats survived the last dose of cocaine at the 90 min time point). (**B**) The blood concentrations of cocaine in rats with or without CocH5-Fc(M6) after cumulative doses of 10, 32, and 56 mg/kg cocaine (at the 75 min time point or 15 min after 56 mg/kg cocaine injection), cumulative doses of 10 and 32 mg/kg cocaine (at the 45 min time point or 15 min after 32 mg/kg cocaine), and 10 mg/kg cocaine (at the 5 min time point). (**C**) The blood concentrations of EME in rats with or without CocH5-Fc(M6) after cumulative doses of 10, 32, and 56 mg/kg cocaine (at the 75 min time point or 15 min after 56 mg/kg cocaine injection), cumulative 10 and 32 mg/kg cocaine (at the 45 min time point or 15 min after 32 mg/kg cocaine injection), and 10 mg/kg cocaine (at the 5 min time point).

To understand the molecular mechanism of the enzyme for blocking cocaine discriminative stimulus effects in rats, we examined the correlation of cocaine blood concentration and the discriminative stimulus effects under the CocH5-Fc(M6) protection. Rats (n = 6 for the control group or n = 4 for the groups with the enzyme treatment) were injected with 10 mg/kg cocaine always at 0 min time point, 32 mg/kg cocaine at 30 min time point and 56 mg/kg at 60 min time point if any of these dose levels was a component of the cumulative doses. The blood concentrations of cocaine and its hydrolytic metabolite ecgonine methyl esterase (EME) were tested at the threshold enzyme concentrations (Table 1) that can completely block cocaine discriminative stimulus effects in rats when administered 10 mg/kg cocaine (IP), cumulative doses of 10 and 32 mg/kg cocaine, or cumulative doses of 10, 32, and 56 mg/kg cocaine. Figure 7A showed the time-dependent blood cocaine concentration in the control group of rats injected with 10, 32, and 56 mg/kg cocaine without enzyme treatment. The blood concentrations of cocaine reached the peak levels of 1040 nM at 5 min after 10 mg/kg cocaine injection, 6370 nM at 45 min (or 15 min after 32 mg/kg cocaine injection), and 17,990 nM at 75 min (or 15 min after 56 mg/kg cocaine injection). Notably, cumulative doses of 10, 32, and 56 mg/kg cocaine were indeed toxic and lethal; 4 out of 6 rats in the control group died at 15–20 min after the third dosing of cocaine (56 mg/kg). The blood concentrations of cocaine and its major hydrolytic metabolite EME in rats with or without CocH5-Fc(M6) at 75 min after cumulative doses of 10, 32, and 56 mg/kg cocaine injection, at 45 min after cumulative doses of 10 and 32 mg/kg cocaine injection, and at 5 min after 10 mg/kg cocaine injection were analyzed and plotted in Fig. 7B,C. In comparison, in the presence of ~ 28.7 mg/L CocH5-Fc(M6) in blood, the concentration of cocaine in rats was negligible after cumulative doses of 10, 32, and 56 mg/kg cocaine, with ~ 99.8% of cocaine was hydrolyzed at 75 min time point (or 15 min after 56 mg/kg cocaine injection); the blood cocaine concentration decreased from ~ 17,990 to 42 nM and the blood EME concentration increased from 6150 to 61,700 nM. Correspondingly, in the presence of ~ 9.38 mg/L CocH5-Fc(M6) in blood, the blood concentration of cocaine at 45 min time point (or 15 min after 32 mg/kg cocaine injection) was reduced from 6370 to 19 nM and the blood concentration of EME was increased from 2380 to 34,740 nM after cumulative doses of 10 and 32 mg/kg cocaine injection, and in the presence of ~ 2.07 mg/L CocH5-Fc(M6) in blood, the blood concentration of cocaine at 5 min was reduced from 1040 to 30 nM and the blood concentration of EME was increased from 483 to 6590 nM after 10 mg/kg cocaine injection. Clearly, CocH5-Fc(M6) rapidly and thoroughly metabolized cocaine to inactive metabolite EME and consequently eliminated interoceptive effects of cocaine, which explains the behavioral effects of CocH5-Fc(M6) observed in DD tests.

## Discussion

In our previous cocaine overdose studies, we demonstrated that 1 mg/kg CocH5-Fc(M6) (IV) powerfully rescued rats from death within a few minutes after the rats were administered a lethal dose of 180 mg/kg cocaine (IP) by rapidly hydrolyzing cocaine in blood; note that even a lower dose of 100 mg/kg cocaine (IP) had 100% lethality (LD_100_) in rats^74,75^. In this study, we further report the prevention effects of recombinant protein CocH5-Fc(M6) on cocaine’s brain actions using a drug discriminative model, demonstrating that CocH5-Fc(M6) is not only highly effective at intercepting and eliminating the psychostimulant effects induced by cocaine, but also capable of blocking the effects for a long period of time. To evaluate the effects of CocH5-Fc(M6) in cocaine discrimination, cocaine doses of 0.32, 1.0, 3.2, 10, 32, and 56 mg/kg were used in our cocaine discrimination tests in comparison with the locomotor activity at cocaine doses of 3.2, 10, 32, and 56 mg/kg, demonstrating that the discriminative stimulus effect of cocaine is more sensitive than the cocaine-induced hyperactivity for detection of cocaine effects. This conclusion was also supported by our previous results that the plasma enzyme concentration for blocking locomotor activity induced by 60 mg/kg cocaine (IP) was 4.4–7.7 mg/L^57^, whereas the average threshold plasma enzyme concentration for blocking cocaine discriminative stimulus effects caused by cumulative 10 and 32 mg/kg cocaine (IP) was ~ 9.38 mg/L. The highest dose of 3 mg/kg CocH5-Fc(M6) was chosen to be consistent with the dose that our lab used in previous animal studies for comparison^54,57^, and two lower enzyme doses 0.3 and 1.0 mg/kg were used to verify dose-dependent effects. The long-term cocaine discrimination tests showed that 0.3, 1.0, and 3.0 mg/kg CocH5-Fc(M6) indeed dose-dependently blocked the discriminative stimulus effects produced by different cumulative doses of cocaine (10, 32, and 56 mg/kg). Particularly, a dose of 3 mg/kg CocH5-Fc(M6) effectively attenuated discriminative stimulus effects of 10 mg/kg cocaine, cumulative doses of 10 and 32 mg/kg cocaine, and cumulative doses of 10, 32 and 56 mg/kg cocaine by ≥ 20% for 41, 19, and 10 days, respectively. So, administration of 3 mg/kg CocH5-Fc(M6) resulted in nearly exclusive responding on the saline-appropriate lever with daily cumulative doses of 10, 32, and 56 mg/kg cocaine, cumulative doses of 10 and 32 mg/kg cocaine, or 10 mg/kg cocaine for 5, 13, or 30 days, respectively. The corresponding minimum enzyme concentrations required to completely block the effects were 221, 72.2 and 15.9 nM, resulting in negligible blood cocaine concentrations. As well-known, 10 mg/kg cocaine produces a blood cocaine peak concentration of 1–3 µM in rats (a common blood concentration in cocaine-dependent users)^27,76^. The sum of cumulative cocaine doses of 10, 32, and 56 mg/kg is 98 mg/kg, and 4 out of 6 rats (~ 67%) died after receiving the final dose (56 mg/kg) of cocaine.

There were two types of enzymes reported previously to study their effects in cocaine discrimination test with rats, one is a mutant of bacterial cocaine esterase (CocE) designed computationally in our Lab^73,77–80^, another one is our earlier version of human BChE mutants^54^. Native CocE itself has a very short half-life in rodents—only a few minutes at physiological temperature (37 °C). Thermally stable CocE mutants were designed, discovered and developed to increase its thermostability in vitro, but the in vivo half-life in rodents was still short^73,78^. Later, Collins et al. reported a PEGylated CocE mutant (G4C/S10C/T172R/G173Q mutant of CocE), known as PEG-CCRQ CocE^73^ to overcome the weakness. Based on their results, 32 mg/kg PEG-CCRQ CocE completely blocked the discriminative effects produced by training dose of 10 mg/kg cocaine for up to 72 h and 3.2 mg/kg or 10 mg/kg PEG-CCRQ CocE resulted in up to 48 h of complete blocking. The duration of CocE’s impact on the discriminative stimulus effects of cocaine was clearly dose dependent. However, the PEG-CCRQ CocE’s blocking effects were still relatively short, lasting for up to 4 days even with the highest dose of 32 mg/kg. In 2020, we reported the blocking effects of CocH3-Fc(M6) in cocaine drug discrimination study^54^. CocH3-Fc(M6) has a biological half-life about 206 ± 7 h in rats^56^. CocH3-Fc(M6) at the same of 3 mg/kg was injected in rats that had stably discriminated cocaine from saline, and it was found that 3 mg/kg CocH3-Fc(M6) completely blocked the discriminative stimulus effects of 10 mg/kg cocaine for 25 days, shorter than 30-day complete blocking provided by 3 mg/kg CocH5-Fc(M6). So, CocH5-Fc(M6) is currently the most effective enzyme against cocaine. With the superior efficacy in blocking cocaine discriminative stimulant effects, CocH5-Fc(M6) is a more promising for continuous development as a pharmacotherapy, satisfying the once-weekly dosing regimen for CUD.

In addition, we also administered 3 mg/kg CocH5-Fc(M6) in rats to test possible effects of CocH5-Fc(M6) on the motivation for food consumption for 32 days. We did not find any significant changes in food responding behavior after the CocH5-Fc(M6) injection. During the entire study (which is as long as 46 days) after the enzyme injection, no adverse signs or abnormal behaviors in rats were observed, indicating that CocH5-Fc(M6) was lacking long-term toxicity in rats. This study provided a crucial profile of CocH5-Fc(M6) with respect to both the potency and duration of the enzyme for prevention of cocaine brain actions by testing its effects against cocaine discriminative stimulus in rats.

## Materials and methods

### Subjects and drugs

Male Sprague–Dawley rats (250–270 g) were ordered from Harlan (Harlan, Indianapolis, IN) and housed individually during the study. All rats were maintained in a temperature- and humidity-controlled environment in the Division of Laboratory Animal Resources at the University of Kentucky. All experimental procedures were approved by the IACUC (Institutional Animal Care and Use Committee) at the University of Kentucky and performed in accordance with the Guide for the Care and Use of Laboratory Animals from National Institutes of Health/Office of Laboratory Animal Welfare (NIH/OLAW), and were in fact also consistent with the ARRIVE (Animal Research: Reporting of In Vivo Experiments) guidelines (https://arriveguidelines.org). All rats had free access to tap water and received a limited amount of rodent food per day to maintain 80% of the daily free-feeding body weights except during testing time.

(-)-Cocaine hydrochloride was obtained from the National Institute on Drug Abuse (NIDA) Drug Supply Program (Bethesda, MD). (+)-Methamphetamine hydrochloride (METH) was purchased from Sigma. All drugs were dissolved in physiologic saline as salt form and administered intraperitoneally (IP) with injection volume 1 ml/kg for drug discrimination study. Food pellets (45 mg/each pellet) were ordered from Bio-Serv (#F0021). Recombinant enzyme CocH5-Fc(M6), a highly efficient long-acting CocH entity to accelerate cocaine metabolism, was designed and prepared by our lab^57^ and administered by intravenous (IV) route.

### Apparatus

Food pellet intake study and cocaine discrimination study were conducted in the same set of operant conditioning chambers ordered from Med Associates, Inc. described in our previous paper^81^. Briefly, each chamber having an interior size of 30.5 cm (L) × 24.1 cm (W) × 21 cm (H) was equipped with two retractable steel response levers and a pellet dispenser in between, which were connected to software MED-PC to program experiments and collect data. Each chamber coupled with a food pellet dispenser and cooperated with lever responding to deliver food pellets through a 5 cm × 4.2 cm opening located on the steel panel of the chamber. A 2.5-cm diameter, yellow stimulus light was centered 4 cm above each response lever and a white house-light was located on the top of the opposite wall.

### CocH5-Fc(M6) plasma concentration analysis

After the enzyme injection, 50–80 μL blood was collected into a heparin-coated capillary tube by saphenous vein puncture. Plasma was separated from the blood samples by centrifuging 15 min at the speed of 5000 g and was kept at 4 °C until analysis. A sensitive radiometric assay^47,54^ with 100 μM [^3^H]-labeled (−)-cocaine was used to analyze the plasma enzymatic activity, which then was converted into the concentration of the enzyme by using the equation [E] = V_max_/*k*_cat_.

### Impact of CocH5-Fc(M6) on response to food pellets

8 rats were trained to earn food pellets in 40-min sessions under fixed ratio (FR) 1 with a 5 s time out (TO) schedule (FR1TO5). The maximum number of rewards (food pellets) allowed in each session was 40. That is, the session would terminate after the maximum of 40 rewards had been earned or 40 min had been elapsed, whichever came first. After the rats acquired stable responding for food pellet, they were administered a single dose of 3.0 mg/kg CocH5-Fc(M6) IV on day 0, and then the testing sessions were performed on days 0, 2, 4, 6, 8, 12, 16, 20, 24, 28, and 32. The test on day 0 was performed at 4 h after the enzyme injection to avoid the potential influence on behavior caused by discomfort (tail pain) through IV injection. The numbers of rewards and response rate (resp/min) were recorded for each session.

### Cocaine drug discrimination study

#### Acquisition of cocaine discrimination behavior

The DD protocol described by Collins et al.^73^ was used as a reference to establish acquisition of cocaine discrimination behavior in rats. Drug discrimination sessions were conducted in operant conditioning chambers (Med Associates, Fairfax, VT). Rats were maintained at 80% of their free-feeding weight for the period of the study. Rats were initially trained to respond under a FR1TO0 schedule for food pellets with both levers active in daily 10-min session (maximum 10 rewards). The FR and TO requirement for pellets was gradually increased over several sessions to a terminal FR10TO5. Completion of a ratio on ether lever resulted in the delivery of a food pellet and resetting of the ratio required on both levers to FR10. Once rats reliably earned a maximum limit of 10 pellets under FR10TO5, they were trained to discriminate 10 mg/kg cocaine (C) from 1 ml/kg saline (S) using a single-alternation (with pattern SCSCS or CSCSC) one-active lever discrimination procedure with responding on the inactive lever was recorded but had no programmed consequence. Lever assignments for each group of rats were alternated and counterbalanced (e.g., the first animal in the group was assigned left-side lever as cocaine-appropriate lever and right-side lever as saline-appropriate lever; second animal in the group was assigned right-side lever as cocaine-appropriate lever and left-side lever as saline-appropriate lever, etc.). For each rat, the assignment would stay fixed throughout the entire study. Following cocaine or saline administration, each rat was placed into an operant chamber illuminated by the house light for a 15-min pretreatment period. The start of the session was programmed and signaled by the offset of the house light, onset the yellow stimulus light above the active lever, and insertion of the levers automatically. Sessions terminated after 10 min had elapsed or 10 food rewards had earned, whichever came first. After rats responded consistently under single alternation schedule, a double-alternation schedule procedure (i.e., CCSSCC or SSCCSS) with the position of cocaine-appropriate lever identical was implemented. The training continued until there were at least seven consecutive sessions during which rats completed at least 80% of responding on the appropriate lever and no more than three responses occurred on the inappropriate lever during the first trial or first ratio (criteria).

Once rats met discrimination criteria, three types of tests were performed: cocaine dose–response curve test, pretreatment of CocH5-Fc(M6) on cocaine dose–response curve test, and long-term effects of CocH5-Fc(M6) on the discriminative stimulus effects of cocaine. Specifically, during all testing sessions, both levers were inserted and active, and both yellow stimulus lights above the levers were illuminated during each component of the session.

#### Cocaine dose–response curve test

To test cocaine dose–response curve, increasing doses of cocaine (0.32, 1.0, 3.2, and 10 mg/kg) were administered (IP) sequentially in a 4-components session (one component for each dose) in eight rats who met the discrimination criteria. The testing doses were chosen based on previously published study^71,73^. Saline session could be administered in initial component of the session or not, thus daily sessions comprised 1–5 components with each component lasting 25 min. METH (0.56 mg/kg) was tested on another day as control to see whether it could substitute cocaine discriminative stimulus effects and maintain lever press behavior in well-trained rats. All drugs were injected as 15-min pretreatments followed by 10 min DD tests in the 25-min components. The time between any two injections was 30 min.

#### Impact of CocH5-Fc(M6) on cocaine-driven interoceptive effects

In five rats who fulfilled discrimination criteria, a low dose of 0.3 mg/kg CocH5-Fc(M6) was injected 4 h before discrimination test as pretreatment to examine the impacts of the enzyme on cocaine-driven interoceptive effects of cocaine with a session comprised of 4 continuous components (0.32, 1.0, 3.2, and 10 mg/kg cocaine) as described above.

#### Long-term effects of CocH5-Fc(M6) on the discriminative stimulus effects of cocaine

To test the long-term effects of CocH5-Fc(M6) on discriminative stimulus effects of cocaine, a single dose of CocH5-Fc(M6) (0.3, 1.0, or 3.0 mg/kg; IV) was injected 4 h before the first component of the drug discrimination session on day 0 in three different groups (n = 6–7/group). Cocaine discrimination tests with cumulative doses of 10, 32, and 56 mg/kg cocaine (IP) were carried out daily until rats allocated at least 80% of their responding on the cocaine-appropriate lever following that dose, after 3–5 days of confirmation, the component of that dose was ceased, while the daily discrimination tests would not stop until 10-mg/kg cocaine training dose produced more than 80% of responding on the cocaine-appropriate lever. In addition, to validate and maintain the standard behavior of lever responding during the long-term testing, administration of 0.56 mg/kg METH occurred in the last component of the daily session every 2 or 3 days. That is, each session was comprised of up to 4 components (in the order of 10, 32, and 56 mg/kg cocaine, followed by 0.56 mg/kg METH) with each component lasting 25 min throughout the whole study. Blood samples were collected from each rat after daily session and measured by the sensitive radiometric assay mentioned above to track the plasma enzyme concentration.

### Cocaine locomotor activity test

To further characterize the effects of cocaine and validate the doses of cocaine used in the study, eight rats were administered 3.2, 10, 32, and 56 mg/kg cocaine (IP) to monitor cocaine-induced hyperactivity and toxicity by using ANY-maze video-tracking system (San Diego Instruments, San Diego, CA) as previous described^82^. The high-density, non-porous plastic testing chambers measuring 50 cm (L) × 50 cm (W) × 38 cm (H) in a light- and sound- attenuating enclosure were used. Rats were put in the chambers for several hours to acclimate the environment. On testing day, before each cocaine or saline administration, rats were allowed to acclimate to the test chamber for 1 h. After cocaine or saline administration, rats were immediately returned to the test chambers for activity monitoring for 3 h. The cumulative distance traveled was recorded in 5-min bins. Before each cocaine test, a locomotor activity baseline was generated by injecting rats with saline. Only one dose of saline (or cocaine) was administered on each test day. The rats took one day off without testing after being tested with any dose of cocaine (3.2, 10, 32, or 56 mg/kg, IP).

### Cocaine metabolism after CocH5-Fc(M6) treatment

Beyond the behavioral test to examine the effectiveness and duration of CocH5-Fc(M6) in blocking interoceptive effects of cocaine by performing cocaine discrimination tests in rats, we also conducted a pharmacodynamic experiment to map the effects of long-acting cocaine hydrolase CocH5-Fc(M6) on cocaine metabolism to understand the behavioral outcomes. Cocaine and EME concentrations were analyzed targeting when the plasma CocH5-Fc(M6) at the threshold levels listed in Table 1: i.e., 28.7, 9.38, and 2.07 mg/L which can completely block cocaine discriminative stimulus effects in rats after injected with highest dose set (cumulative 10, 32, and 56 mg/kg cocaine, IP), middle dose set (cumulative 10 and 32 mg/kg cocaine, IP), and lowest dose set (10 mg/kg cocaine, IP), respectively.

Rats (n = 6) in the control group were injected the doses of 10, 32, and 56 mg/kg cocaine at 0, 30, and 60 min, respectively, blood samples (~ 75 μL) were collected from saphenous veins into heparin-treated capillary tubes at various time points after injection of (−)-cocaine and mixed immediately with 100 μL of paraoxon (25 μM in 0.1% formic acid). Blood samples were stored at − 80 °C until analysis using our previously reported LC–MS/MS method^83^ for simultaneously determining the concentrations of (−)-cocaine and its metabolite ecgonine methyl ester (EME) in blood samples. Briefly, our LC–MS/MS protocol includes a solid-phase extraction that is a single-step process capable of extracting cocaine and cocaine metabolites from the blood samples^83^. A Shimadzu HPLC system (Shimadzu, Kyoto, Japan) consisting of a DGU-20A/3R degasser, LC-20AD binary pumps, CBM-20A controller, and SIL-20A/HT auto sampler, was used. Chromatographic analysis was performed on an Atlantis T3 (100 Å, 3 μm, 2.1 mm × 150 mm I.D) column (Waters, Milford, MA). A mass spectrometer, AB SCIEX triplet OF 5600 (Redwood City, CA), was run in positive ion and high sensitivity mode, with positive ions generated in the source using nitrogen as the source gas.

To test the enzyme effects, rats (n = 4) were administered with 0.7 mg/kg CocH5-Fc(M6) via IV, and the plasma samples were collected at various time points to monitor the CocH5-Fc(M6) concentrations in the plasma. When the enzyme concentration became close to ~ 28.7 mg/L (at 28.6 ± 0.50 mg/L on day 0) in the plasma, rats were injected the cumulative doses of 10, 32, and 56 mg/kg cocaine at 0, 30 and 60 min respectively, and blood samples were collected at 75 min for cocaine and EME analysis. When the plasma enzyme concentration decreased to roughly ~ 9.38 mg/kg (at 11.3 ± 1.2 mg/L on day 2), cumulative doses of 10 and 32 mg/kg cocaine were respectively injected at 0 and 30 min and blood samples were collected at 45 min for cocaine and EME analysis. Another group of 4 rats (n = 4) were administered 0.1 mg/kg CocH5-Fc(M6) via IV. When the enzyme concentration was close to ~ 2.07 mg/L (at 2.5 ± 0.1 mg/L on day 0) in the plasma, rats were injected with 10 mg/kg cocaine at 0 min and blood samples at 5 min were collected for blood cocaine and EME analysis.

### Data analysis

All data are presented as mean ± the standard error of the mean (SEM) by using GraphPad Prism 9 (Software, San Diego, CA). Two sets of information were extracted from cocaine discrimination study, one is overall responding (lever presses) that occurred on the cocaine-appropriate lever (% cocaine lever resp), another one is the overall response rate (resp/second) during the active portion of each component. A complete blocking of the discriminative stimulus effects of cocaine was defined as less than 20% of the overall responding occurring on the cocaine-appropriate lever during any component after enzyme injection; a full generalization to the discriminative stimulus effects of cocaine was defined as more than 80% of overall responding occurring on the cocaine-appropriate lever during any component, which also indicated that enzyme could not block cocaine discriminative stimulus effects any longer at that time of point. To clearly visualize cocaine-induced responding, two dot lines were drawn at 20% and 80% cocaine levels responding in all cocaine lever responding figures in this report. The lowest enzyme concentration which completely blocked discriminative effects induced by a specific dose of cocaine (with less than 20% of cocaine lever responding) was defined as the threshold enzyme concentration for that dose of cocaine.

One-way ANOVA with post hoc Dunnett's test was used to analyze significance in cocaine-dose response curve test, and two-way ANOVA with post hoc test was conducted to analyze significance in cocaine-driven interoceptive effects of cocaine (cocaine dose–response curve) with or without enzyme injection with post hoc Bonferroni's multiple comparisons test. Two-way repeated measure ANOVA and one-way ANOVA with post hoc Tukey's multiple comparisons test were used to analyze distance moved in 5 min and total distance moved in 3 h after injection, respectively, in locomotor activity study. Statistical significance was set at *p* < 0.05.

## Data Availability

The datasets used and/or analyzed during the current study are available from the corresponding author on reasonable request.
